# The incidence of chronic diarrhea decreases with increasing serum calcium levels: a cross-sectional study based on NHANES 2005–2010

**DOI:** 10.1186/s12876-023-03029-2

**Published:** 2023-11-15

**Authors:** Xiaotong Li, Jiali Li, Zhiqun Cao, Nan Kang

**Affiliations:** 1 The First Clinical College, Shandong University of Traditional Chinese Medicine, No. 4655 University Road, University Science Park, Changqing District, Jinan, Shandong Province 250355 China; 2 Department of Traditional Chinese Medicine, Affiliated Hospital of Jining Medical University, No. 89, Guhuai Road, Rencheng District, Jining, Shandong Province 272029 China; 3 Department of Gastroenterology, Affiliated Hospital of Shandong University of Traditional Chinese Medicine, No. 42 Wenhua West Road, Jinan, Shandong Province 250011 China

**Keywords:** Cross-sectional study, Serum calcium, Chronic diarrhea, NHANES

## Abstract

**Background:**

Chronic diarrhea is difficult to prevent and treat due to its complex etiology and pathogenesis. It places a huge burden on patients and public healthcare. It is known that the regulation of body homeostasis relies heavily on calcium. However, in the general population, the relationship between calcium and chronic diarrhea remains uncertain.

**Methods:**

We assessed the association between serum calcium and diarrhea using data from the 2005–2010 National Health and Nutrition Examination Survey (NHANES). Serum calcium level was measured from collected blood samples. Diarrhea was assessed using the Bristol Stool Scale (BSFS) (types 1–7). The stability of the results was assessed using logistic regression and sensitivity analysis. The dose–response association between serum calcium and the risk of diarrhea was analyzed using a restricted cubic spline plot.

**Results:**

This study included 12,342 participants. In each of the five models, an increased calcium level was negatively associated with the incidence of diarrhea (OR[95%CI]:0.26 [0.13–0.53], 0.28 [0.14–0.58], 0.4 [0.19–0.82], 0.27 [0.11–0.64] and 0.24 [0.10–0.59], respectively). When serum calcium was analyzed as a categorical variable, a significant association between serum calcium and diarrhea prevalence was found. The restricted cubic spline plot showed a linear relationship between serum calcium and diarrhea. Sensitivity analysis confirmed that the results were stable.

**Conclusion:**

The results of our cross-sectional study suggest that a higher level of serum calcium may reduce the incidence of diarrhea. In the future, this finding should be further validated in a randomized controlled trial.

**Supplementary Information:**

The online version contains supplementary material available at 10.1186/s12876-023-03029-2.

## Introduction

Diarrhea, defined as loose stools and more frequent bowel movements, is a common problem in gastroenterology. Most diarrhea is acute and self-limiting, with acute diarrhea usually resolving within 4 weeks in most patients [1]. Chronic diarrhea is defined as passing loose stools 3 or more times a day for at least 4 weeks [2, 3]. Research studies have shown that the prevalence of chronic diarrhea is between 14 and 18% in the United States [4]. Chronic diarrhea is not usually infectious, but the differential diagnosis remains complex. Yu et al. [5] found that among patients under 60 years of age, chronic diarrhea or constipation was positively correlated with breast cancer, colon cancer, cardiovascular disease, all-cause mortality risk and cardiovascular disease mortality.

Calcium is the most common mineral element in the human body [6] and is involved in a wide range of biological activities. As an intracellular second messenger, calcium is essential in regulating homeostasis, especially water and electrolyte absorption and secretion in the gastrointestinal tract [7]. Elevated intracellular calcium ion concentrations can reduce the absorption of potassium and sodium ions, increase the secretion of chloride ions and elevate the electrolyte content in the intestinal lumen, leading to diarrhea. Although a high intracellular calcium level contributes to the development of diarrhea, the relationship between the extracellular calcium level and diarrhea remains unknown. Some investigators have found that a higher serum calcium level is significantly associated with decreased survival in colorectal cancer [8]. However, whether serum calcium affects diarrhea remains to be explored.

About 50% of the body's calcium exists in the serum as an ion, 40% is bound to albumin, and the remaining 10% is bound to anions [9]. Total serum calcium refers to the sum of the three forms, and is independent of any physiological or quantitative changes. Clinically total serum calcium is often used to indicate the amount of calcium in the body [6]. Therefore, in this secondary cross-sectional study, using data from a substantial and representative American population, we examined the relationship between total serum calcium and diarrhea.

## Methods

### Study population and data sources

This cross-sectional study utilized continuous data from the National Health and Nutrition Examination (NHANES) study from 2005 to 2010. Our study included participants aged 20 years and older, who completed interviews and exams at designated mobile examination centers (MECs). Participants with gut health problems, missing serum calcium result and missing covariate data were excluded. NHANES is a health screening program that uses a multi-level probability model to select respondents for a representative sample, aiming to assess the health and nutritional status of American citizens [10]. Staff conducted extensive household interviews to collect demographic data and health histories.. At the MEC, a physical examination is performed and a blood sample is taken. The CDC Laboratory Science Division analyzed the serum samples obtained from the National Center for Environmental Health.

Participants provided informed consent to participate in the NHANES study, which was approved by the National Health Statistics Ethics Review Board. The initial study protocol is available on the National Institutes of Health Statistical Review Board website (https://www.cdc.gov/nchs/nhanes/irba98.htm). The study was formally authorized by the Institutional Review Board. (Protocol No. 2005–06). Research data are freely available to researchers worldwide. As this is a secondary cross-sectional analysis, it did not require additional approval from the institutional ethics review committee.

### Measurement of serum calcium

Serum calcium was measured from blood samples collected at the MEC. The collected samples were stored at -30 °C, until they ware sent to the National Centre for Environmental Health for analysis. Detailed specimen collection and processing instructions from the NHANES Laboratory/Medical Technician Procedures Manual (LPM) were followed. In this study, serum calcium was analyzed as a continuous variable.

### Bowel health questionnaire and the definition of chronic diarrhea

The Gut Health Questionnaire was completed in the MEC survey room using a computerized personal survey system. The Bristol stool scale, commonly used in research and clinical practice, was used to define chronic diarrhea [11]. Previous studies have demonstrate that individual stool results are strongly correlated with gastrointestinal transit time measurements of radiopaque markers [12].

Participants were shown a card with color pictures and descriptions of the seven Bristol stool types (types 1 to 7). Those who correlated their regular stool as being most similar to stool type 6 (fluffy stool with rough edges and a moist appearance) or type 7 (watery, non-solid stool) were classified as having chronic diarrhea [12, 13].

### Covariates

Age, sex, race, marital status, educational level, household income, smoking, alcohol abuse, albumin, serum iron, cholesterol, total protein, uric acid, serum vitamin D, sodium, potassium, and hemoglobin were analyzed in this study. Calcium intake, body mass index (BMI), diabetes, arthritis, renal failure, osteoporosis, thyroid disease, and physical activity were listed as possible confounding factors. Race and ethnicity were grouped into Mexican–American, Other Hispanic, Non-Hispanic White, Non-Hispanic Black and Other. Education level was divided into those without a high school diploma and those with at least a high school education. Marital status was classified as married, widowed/divorced and never married. Household income was divided into high earners and low earners, using a $20,000 threshold.

The Beckman Synchron LX20 analyzer was used to detect serum albumin (g/L), serum iron (μmol/L), serum cholesterol (mmol/L), serum phosphorus (mmol/L), total protein (g/L), uric acid (μmol/L), blood sodium (mmol/L), blood potassium (mmol/L). Analysis of 25-hydroxyvitamin D3 [25(OH)D3], 25-hydroxyvitamin D2 [25(OH)D2] and 3-epi serum samples by high performance liquid chromatography tandem mass spectrometry (UHPLC-MS/MS)- 25- Concentration of hydroxyvitamin D3 [3-epi-25(OH)D3]. Total vitamin D status was defined as the sum of 25(OH)D3 and 25(OH)D2. Hemoglobin (g/dL) was based on a Beckman Coulter counter calibration method using an automatic dilution mixer for sample processing and measured by a single-beam photometer.

Calcium intake was obtained from the MEC ​​food recall interview, and dietary information was collected 24 h before the interview. BMI was calculated from the measured height and weight, and expressed in kilograms per square meter. Weight was measured on a digital scale in pounds and converted to kilograms. Altitude was measured with an electronic altimeter, accurate to the millimeter.

Smoking status was categorized into two groups: participants who smoked 100 or more cigarettes during their lives were described as ≥ 100 cigarettes during their lifetime, those who smoke less than 100 cigarettes in their lifetime are considered to be non-smokers. Those who consume at least 12 alcoholic drinks annually were defined as drinkers [14]. Diabetes, arthritis, kidney disease, thyroid disease and osteoporosis were defined according to participant to the following questions: "Have doctors or other health professionals ever told you that you have diabetes, arthritis, kidney disease, thyroid disease or osteoporosis?" High intensity exercise was defined as at least 10 min of vigorous activity (exercises that resulted in profuse sweating and a significant increase in respiratory or heart rates) in the past 30 days. Moderate intensity exercise was defined as at least 10 min of physical activity (exercises that resulted in light sweating or a mild to moderate increase in respiratory or heart rates) during the past 30 days.

### Statistical analysis

All analyses were performed using the R statistical package (http://www.R-project.org, R Foundation) and, Free Statistical software, version 1.7.1 [15]. Demographic and clinical data are described using means ± standard deviations and frequencies (percentages). Data that followed a normal distribution were analyzed using the t-test, while non-parametric continuous variables were analyzed using the Kruskal–Wallis test. In multiple logistic regression, serum calcium was analyzed as both a continuous and categorical variable. Model 1 adjusted for age, gender, race, education, marital status, and household income, Model 2 adjusted for model 1 variables as well as including smoking status, drinking, high intensity exercise, moderate intensity activity, and body mass index, and Model 3 adjusted for model 2 as well as serum albumin, serum iron, serum cholesterol, serum phosphorus, serum total protein, uric acid, serum vitamin D, serum sodium, serum potassium, hemoglobin, and calcium intake. Model 4 adjusted for model 3 as well as diabetes, arthritis, renal failure, osteoporosis, thyroid disease, and depression. A sensitivity analysis was performed by excluding outliers with serum calcium outside the mean ± 2SD (0–2.6 mmol/L) or mean ± 3SD (0–2.7 mmol/L) ranges. *P* value < 0.05 was considered statistically significant. The dose–response association between serum calcium and risk of diarrhea was analyzed using a restricted cubic spline plot.

## Results

### Baseline characteristics of the study population

**Fig. 1:**
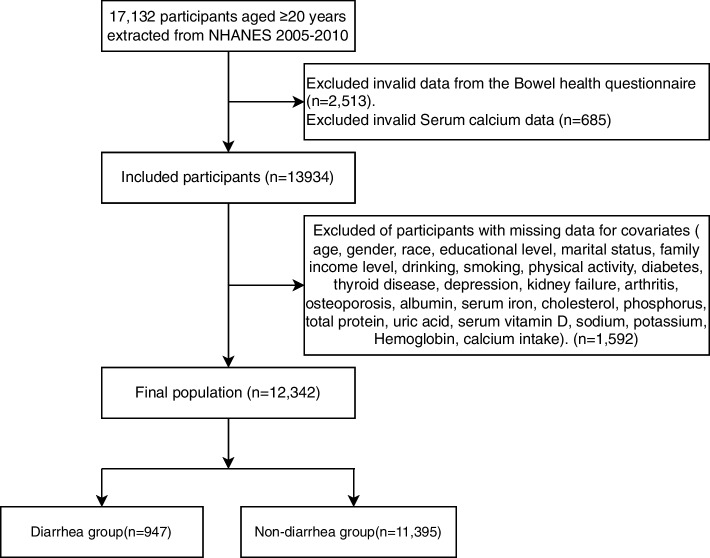
The study’s flow diagram

This study recruited 31,034 potential NHANES participants (2005–2010), of whom 17,132 adults (aged 20 years or older) completed interviews and were screened by the MEC for inclusion in our study. Participants who did not respond to the gut health and serum calcium questionnaires were excluded (*n* = 3,198). After eliminating participants with missing covariate data (*n* = 1592), 12,342 participants were included in the final analysis. Figure 1 shows a flowchart of the exclusion criteria. Table 1 shows the baseline characteristics of patients with diarrhea. In our study, diarrhea was more likely in participants who were male, non-Hispanic white, less educated, smoker, alcoholic, hypertensive, and those with little work or leisure time. Daily dietary intakes of serum albumin, serum calcium, serum iron, serum vitamin D, hemoglobin, uric acid, and calcium were significantly lower in participants with diarrhea compared with participants without diarrhea. Participants in the diarrhea group were characterized by their older age and high BMI.

**Table 1:** Characteristics of Participants in the NHANES 2005–2010

Variables	Total (*n* = 12342)	Non-diarrhea (*n* = 11395)	Diarrhea (*n* = 947)	*p*-value
Gender, n (%)				< 0.001
Female	6284 (50.9)	5743 (50.4)	541 (57.1)	
Male	6058 (49.1)	5652 (49.6)	406 (42.9)	
Age (year), Mean ± SD	49.2 ± 17.9	48.9 ± 18.0	53.4 ± 16.5	< 0.001
Race, n (%)				< 0.001
Mexican American	2238 (18.1)	2027 (17.8)	211 (22.3)	
Other Hispanic	968 (7.8)	881 (7.7)	87 (9.2)	
Non-Hispanic White	6325 (51.2)	5902 (51.8)	423 (44.7)	
Non-Hispanic Black	2338 (18.9)	2147 (18.8)	191 (20.2)	
Other Race	473 (3.8)	438 (3.8)	35 (3.7)	
Education, n(%)				< 0.001
< 12^th^ Grad	6293 (51.0)	5703 (50)	590 (62.3)	
High School Grad/GED or higher	6049 (49.0)	5692 (50)	357 (37.7)	
Marital status, n (%)				< 0.001
Married	6694 (54.2)	6171 (54.2)	523 (55.2)	
Widowed/Divorced	2306 (18.7)	2091 (18.4)	215 (22.7)	
Never married	3342 (27.1)	3133 (27.5)	209 (22.1)	
Family income ($), n (%)				< 0.001
< 20,000	2975 (24.1)	2677 (23.5)	298 (31.5)	
≥ 20,000	9367 (75.9)	8718 (76.5)	649 (68.5)	
Smoking status, n (%)				0.004
Non-smoker	6461 (52.3)	6008 (52.7)	453 (47.8)	
≥ 100 cigarettes during their lifetime	5881 (47.7)	5387 (47.3)	494 (52.2)	
Drinking, n (%)				0.001
No	3476 (28.2)	3166 (27.8)	310 (32.7)	
Yes	8866 (71.8)	8229 (72.2)	637 (67.3)	
Albumin (g/L) Mean ± SD	42.1 ± 3.6	42.1 ± 3.6	41.6 ± 3.6	< 0.001
Serum calcium (mmol/L), Mean ± SD	2.4 ± 0.1	2.4 ± 0.1	2.3 ± 0.1	< 0.001
Cholesterol (mmol/L), Mean ± SD	5.1 ± 1.1	5.1 ± 1.1	5.1 ± 1.1	0.95
Serum iron (μmol/L), Mean ± SD	15.3 ± 6.4	15.3 ± 6.4	14.6 ± 6.2	< 0.001
Phosphorus (mmol/L), Mean ± SD	1.2 ± 0.2	1.2 ± 0.2	1.2 ± 0.2	0.652
Total protein (g/L), Mean ± SD	71.4 ± 4.9	71.4 ± 4.8	71.8 ± 5.3	0.016
Uric acid (μmol/L), Mean ± SD	324.5 ± 86.5	323.9 ± 86.1	332.0 ± 90.4	0.005
Sodium (mmol/L), Mean ± SD	139.2 ± 2.3	139.2 ± 2.3	139.0 ± 2.6	0.073
Potassium (mmol/L), Mean ± SD	4.0 ± 0.3	4.0 ± 0.3	4.0 ± 0.4	0.029
Serum Vitamin D (nmol/L), Mean ± SD	61.5 ± 24.4	61.7 ± 24.6	58.6 ± 22.6	< 0.001
Hemoglobin (g/dL), Mean ± SD	14.2 ± 1.5	14.2 ± 1.5	13.9 ± 1.6	< 0.001
Calcium intake(mg), Mean ± SD	805.0 (523.0, 1181.0)	811.0 (527.0, 1186.0)	744.0 (492.0, 1090.0)	< 0.001
Body Mass Index (kg/m^2^), Mean ± SD	29.1 ± 6.7	29.0 ± 6.6	30.8 ± 8.1	< 0.001
Diabetes, n (%)				< 0.001
No	10963 (88.8)	10192 (89.4)	771 (81.4)	
Yes	1379 (11.2)	1203 (10.6)	176 (18.6)	
Depression, n (%)				< 0.001
No	11316 (91.7)	10528 (92.4)	788 (83.2)	
Yes	1026 (8.3)	867 (7.6)	159 (16.8)	
Kidney failure, n (%)				< 0.001
No	12042 (97.6)	11138 (97.7)	904 (95.5)	
Yes	300 (2.4)	257 (2.3)	43 (4.5)	
Arthritis, n (%)				< 0.001
No	8972 (72.7)	8364 (73.4)	608 (64.2)	
Yes	3370 (27.3)	3031 (26.6)	339 (35.8)	
Osteoporosis, n (%)				0.001
No	11655 (94.4)	10783 (94.6)	872 (92.1)	
Yes	687 (5.6)	612 (5.4)	75 (7.9)	
Thyroid disease, n (%)				0.037
No	11146 (90.3)	10309 (90.5)	837 (88.4)	
Yes	1196 (9.7)	1086 (9.5)	110 (11.6)	
High intensity exercise, n (%)				0.002
No	9483 (76.8)	8716 (76.5)	767 (81)	
Yes	2859 (23.2)	2679 (23.5)	180 (19)	
Moderate intensity exercise, n (%)				< 0.001
No	6979 (56.5)	6389 (56.1)	590 (62.3)	
Yes	5363 (43.5)	5006 (43.9)	357 (37.7)	

### Association between serum calcium and diarrhea

A multivariate logistic regression analysis is presented in Table 2. In the unadjusted model, serum calcium was inversely related to diarrhea (OR 0.26; 95% CI 0.13–0.53). The outcome was similar after adjusting for age, gender, race, education, marital status, and household income (OR, 0.28; 95% CI, 0.14–0.58). After adjusting for other potentially confounding factors, including smoking status, drinking, physical activity, BMI, albumin, serum iron, cholesterol, phosphorus, total protein, uric acid, serum vitamin D, sodium, potassium, hemoglobin, calcium intake, diabetes, arthritis, kidney failure, osteoporosis, thyroid disease, depression, the negative association remained significant (all *P* < 0.001). This significant correlation also appeared in several adjusted models when serum calcium was analyzed as a categorical variable.

**Table 2:** Multiple regression modeling of the relationship between serum calcium and the risk of diarrhea

Variable	Cases/participants	Non-adjust		Model 1		Model 2		Model 3		Model 4	
		OR 95% CI	*P*-value	OR 95% CI	*P*-value	OR 95% CI	*P*-value	OR 95% CI	*P*-value	OR 95% CI	*P*-value
Serum calcium	947/12342	0.26 (0.13 ~ 0.53)	< 0.001	0.28 (0.14 ~ 0.58)	0.001	0.4 (0.19 ~ 0.82)	0.012	0.27 (0.11 ~ 0.64)	0.003	0.24 (0.1 ~ 0.59)	0.002
Quartile 1	239/2483	1 (Ref)		1 (Ref)		1 (Ref)		1 (Ref)		1 (Ref)	
Quartile 2	197/2547	0.79 (0.65 ~ 0.96)	0.017	0.81 (0.67 ~ 0.99)	0.042	0.84 (0.69 ~ 1.03)	0.089	0.82 (0.67 ~ 1)	0.055	0.8 (0.65 ~ 0.98)	0.032
Quartile 3	283/4074	0.7 (0.59 ~ 0.84)	< 0.001	0.73 (0.61 ~ 0.88)	0.001	0.77 (0.64 ~ 0.93)	0.006	0.73 (0.6 ~ 0.89)	0.002	0.72 (0.59 ~ 0.89)	0.002
Quartile 4	228/3238	0.71 (0.59 ~ 0.86)	< 0.001	0.74 (0.61 ~ 0.9)	0.002	0.81 (0.67 ~ 0.99)	0.04	0.75 (0.59 ~ 0.94)	0.012	0.72 (0.58 ~ 0.91)	0.006
*p* for trend			< 0.001		0.001		0.024		0.007		0.004

Model 1: adjust age, gender, race, education, marital status, family income

Model 2: model 1 + smoking stuts, drinking, high intensity exercise, moderate intensity activity

Model 3: model 2 + albumin, serum iron, cholesterol, phosphorus, total protein, uric acid, serum vitamin D, sodium, potassium, calcium intake

Model 4: model 3 + diabetes, arthritis, kidney failure, osteoporosis, thyroid disease

### Restricted cubic spline analysis and sensitive analysis

**Fig. 2:**
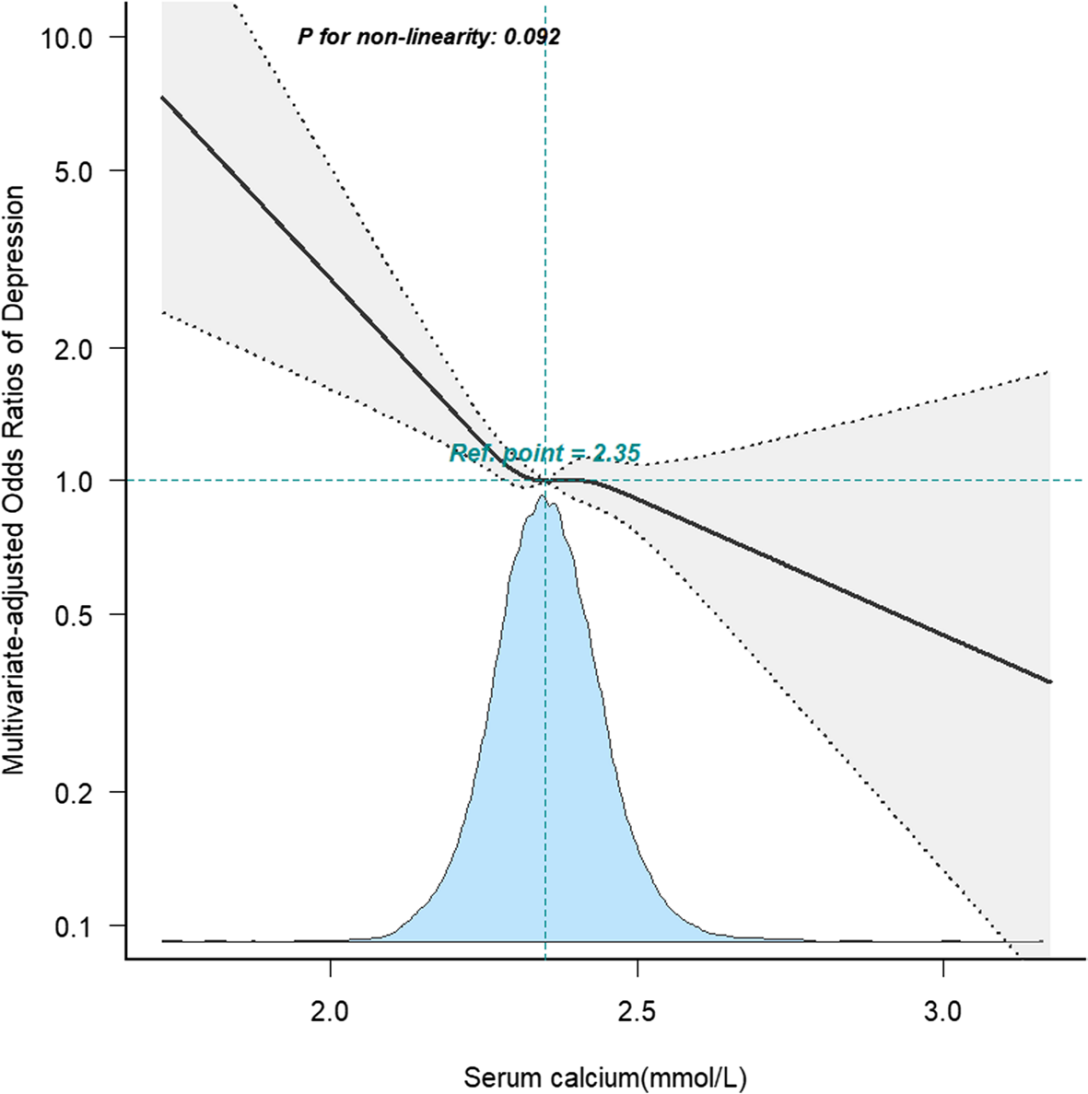
Restricted cubic spline for the serum calcium to diarrhea risk ratio. Adjusted for age, gender, race, education, marital status, family income, smoking stuts, drinking, high intensity exercise, moderate intensity activity, albumin, serum iron, cholesterol, phosphorus, total protein, uric acid, serum vitamin D, sodium, potassium, calcium intake, diabetes, arthritis, kidney failure, osteoporosis, thyroid disease

The dose-response relationships between serum calcium and diarrhea risk was examined using a restrictive cubic spline analysis. Figure 2 shows that a non-linear relationship between serum calcium and the risk of diarrhea does not hold. Result from the sensitivity analysis supported our findings.

## Discussion

In this cross-sectional analysis of data of adults (20 years and older) from NHANES (2005–2010) in the USA, we found that serum calcium was negatively associated with the risk of diarrhea. This study examined the relationship between serum calcium and diarrhea in US adults. Our results demonstrated that high serum calcium levels reduced the risk of diarrhea, which is consistent with findings from several earlier studies [16, 17].

In a previous studies, Cheng et al [16] showed that simple nutritional calcium(Give calcium-rich dairy products or intravenous calcium carbonate) may help to “stop” diarrhea quickly. Similarly, Fraebel et al [17] in a case report of a malnourished child with immune-mediated enteropathy, confirmed that diarrheal symptoms were negatively associated with serum calcium ion level during episodes of the disease. Diarrhea occurred when serum calcium ion was low, while calcium ion levels returned to normal, and diarrhea ceased rapidly.

In recent years, researchers have increasingly focused on serum calcium-related studies. In particular, hypocalcemia was associated with a higher risk of all-cause mortality and cardiovascular disease mortality [18–20]. However, to date, little attention has been paid to the relationship between serum calcium and diarrhea.

Though little is understood about the mechanisms by which serum calcium affects diarrhea, several studies have focused on one key receptor, namely, the calcium-sensitive receptor (CaSR). CaSR is a member of the CG protein-coupled receptor (GPCR) class, which directly regulates renal calcium excretion and indirectly regulates parathyroid hormone (PTH) release from the parathyroid glands, thereby improving calcium homeostasis [21]. CaSR is highly expressed in the mammalian gut by transport epithelial cells, fluid/motor-regulated enteric nerves and intestinal inflammation-regulating cells [22]. Firstly, activated CaSR inhibits anion secretion in the colon and increases aqueous absorption in the intestine [23–25]. Second, activation of the intestinal CaSR reduces overactive intestinal nerve activity and peristalsis, thereby relieving diarrhea [21]. Alternatively, calcium-sensitive receptors can reduce diarrhea by inhibiting intestinal inflammation [26, 27].

In this study, the relationship between serum calcium and diarrhea was investigated after adjusting for baseline characteristics and potential variables. This study has several strengths. First, we included a large nationally representative sample of American adults. Second, we considered and adjusted for known and potential diarrheal risk factors. Third, we performed a dose–response analysis to evaluate the association between different serum calcium levels and diarrhea. In addition, a sensitivity analysis was performed to verify the stability of the results.

The study also has some limitations. First, causal deductions cannot be derived from cross-sectional studies, because causality is temporal in nature, i.e., it must be clear that the cause comes first and the effect follows, whereas the the cause and the over of cross-sectional study is acquired at the same time. Second, the interview data in this study were self-reported. This may pose a risk as questions may be misinterpreted or recalled inaccurately, which could lead to bias. The accuracy of the results may be improved if the same participants had been resampled in different stages of the cycle. However, this was not possible as participants were recruited using a multi-level randomized stratified design and NHANES surveys approximately 5,000 people annually in 15 different districts across the country. Finally, although adjustments were made for various confounding factors, we were unable to exclude the possibility that the observed associations may be due to unmeasured confounding factors. Given these constraints, carefully designed multicenter stochastic controlled trials are needed to confirm our findings.

## Conclusion

Our results suggest that there is a linear inverse relationship between serum calcium and diarrhea. In the future, prospective studies on the causal relationship between serum calcium levels and patients with diarrhea are needed.

### Supplementary Information

**Additional file 1.** Additional results of the sensitivity analysis.

## Data Availability

All data used in this article can be obtained from the National Health and Nutrition Examination Survey(https://www.cdc.gov/nchs/nhanes/index.htm).
