# Supplementary material for: The incidence of chronic diarrhea decreases with increasing serum calcium levels: a cross-sectional study based on NHANES 2005–2010
Source: BMC Gastroenterol. 2023 Nov 15;23:394. doi: 10.1186/s12876-023-03029-2 (PMC10647030; doi:10.1186/s12876-023-03029-2)
Supplement: Supplementary file 1 — Additional file 1. Additional results of the sensitivity analysis. [file 12876_2023_3029_MOESM1_ESM.docx]

**Additional results of the sensitivity analysis**

0-mean+2sd

| Variable | Non-adjust | |  | Model 1 | |  | Model 2 | |  | Model 3 | |  | Model 4 | |
| --- | --- | --- | --- | --- | --- | --- | --- | --- | --- | --- | --- | --- | --- | --- |
|  | OR 95% CI | P-value |  | OR 95% CI | P-value |  | OR 95% CI | P-value |  | OR 95% CI | P-value |  | OR 95% CI | P-value |
| Serum calcium | 0.21 (0.1~0.43) | <0.001 |  | 0.24 (0.11~0.51) | <0.001 |  | 0.35 (0.16~0.74) | 0.006 |  | 0.2 (0.08~0.51) | 0.001 |  | 0.19 (0.08~0.47) | <0.001 |
| Quartile 1 | 1(Ref) |  |  | 1(Ref) |  |  | 1(Ref) |  |  | 1(Ref) |  |  | 1(Ref) |  |
| Quartile 2 | 0.79 (0.65~0.96) | 0.017 |  | 0.81 (0.67~0.99) | 0.041 |  | 0.84 (0.69~1.03) | 0.088 |  | 0.81 (0.66~1) | 0.046 |  | 0.79 (0.65~0.97) | 0.027 |
| Quartile 3 | 0.7 (0.59~0.84) | <0.001 |  | 0.73 (0.61~0.87) | 0.001 |  | 0.77 (0.64~0.93) | 0.006 |  | 0.73 (0.59~0.89) | 0.002 |  | 0.72 (0.59~0.88) | 0.001 |
| Quartile 4 | 0.7 (0.58~0.85) | <0.001 |  | 0.74 (0.61~0.89) | 0.002 |  | 0.81 (0.66~0.99) | 0.036 |  | 0.73 (0.58~0.92) | 0.008 |  | 0.71 (0.57~0.9) | 0.004 |
| p for trend |  | <0.001 |  |  | 0.001 |  |  | 0.02 |  |  | 0.004 |  |  | 0.003 |

0-mean+3sd

| Variable | Non-adjust | |  | Model 1 | |  | Model 2 | |  | Model 3 | |  | Model 4 | |
| --- | --- | --- | --- | --- | --- | --- | --- | --- | --- | --- | --- | --- | --- | --- |
|  | OR 95% CI | P-value |  | OR 95% CI | P-value |  | OR 95% CI | P-value |  | OR 95% CI | P-value |  | OR 95% CI | P-value |
| Serum calcium | 0.26 (0.13~0.55) | <0.001 |  | 0.29 (0.14~0.61) | 0.001 |  | 0.41 (0.2~0.87) | 0.02 |  | 0.25 (0.1~0.61) | 0.002 |  | 0.23 (0.1~0.57) | 0.001 |
| Quartile 1 | 1(Ref) |  |  | 1(Ref) |  |  | 1(Ref) |  |  | 1(Ref) |  |  | 1(Ref) |  |
| Quartile 2 | 0.79 (0.65~0.96) | 0.017 |  | 0.81 (0.67~0.99) | 0.042 |  | 0.84 (0.69~1.03) | 0.088 |  | 0.81 (0.66~0.99) | 0.043 |  | 0.79 (0.64~0.97) | 0.026 |
| Quartile 3 | 0.7 (0.59~0.84) | <0.001 |  | 0.73 (0.61~0.88) | 0.001 |  | 0.77 (0.64~0.93) | 0.006 |  | 0.72 (0.59~0.88) | 0.001 |  | 0.72 (0.59~0.88) | 0.001 |
| Quartile 4 | 0.71 (0.59~0.86) | 0.001 |  | 0.75 (0.62~0.91) | 0.003 |  | 0.82 (0.67~1) | 0.046 |  | 0.74 (0.59~0.92) | 0.008 |  | 0.72 (0.57~0.9) | 0.004 |
| p for trend |  | <0.001 |  |  | 0.001 |  |  | 0.027 |  |  | 0.005 |  |  | 0.003 |

Model 1: adjust age, gender, race, education, marital status, family income.

Model 2: model 1+somking, drinking, high intensity exercise, moderate intensity exercise.

Model 3: model 2+albumin, serum iron, cholesterol, phosphorus, total protein, uric acid, serum vitamin D, sodium, potassium, calcium intake.

Model 4: model 3+diabetes, arthritis, kidney failure, osteoporosis, thyroid disease.
